# Dataset of integrin-linked kinase protein: Protein interactions in cardiomyocytes identified by mass spectrometry

**DOI:** 10.1016/j.dib.2016.03.056

**Published:** 2016-03-19

**Authors:** Alexandra Traister, Mingliang Lu, John G. Coles, Jason T. Maynes

**Affiliations:** a Division of Cardiovascular Surgery, Hospital for Sick Children, Toronto, Ontario, Canada M5G 1X8; b Department of Anesthesia and Pain Medicine, Hospital for Sick Children, Toronto, Ontario, Canada M5G 1X8; c Departments of Anesthesia and Biochemistry, University of Toronto, Toronto, Ontario, Canada M5S 2J7

**Keywords:** Cardiology, Protein:protein interactions, Calcium regulation, Mass spectrometry

## Abstract

Using hearts from mice overexpressing integrin linked kinase (ILK) behind the cardiac specific promoter αMHC, we have performed immunoprecipitation and mass spectrometry to identify novel ILK protein:protein interactions that regulate cardiomyocyte activity and calcium flux. Integrin linked kinase complexes were captured from mouse heart lysates using a commercial antibody, with subsequent liquid chromatography tandem mass spectral analysis. Interacting partners were identified using the MASCOT server, and important interactions verified using reverse immunoprecipitation and mass spectrometry. All ILK interacting proteins were identified in a non-biased manner, and are stored in the ProteomeXchange Consortium via the PRIDE partner repository (reference ID PRIDE: PXD001053). The functional role of identified ILK interactions in cardiomyocyte function and arrhythmia were subsequently confirmed in human iPSC-cardiomyocytes.

**Specifications Table**

**Table:** 

Subject area	*Biology*
More specific subject area	*Molecular Cardiology*
Type of data	*Mass Spectrometry Table*
How data was acquired	*On-line liquid chromatography-tandem mass spectrometry set-up using an Agilent 1100 Capillary LC system fitted to a LTQ ion trap mass spectrometer. A C18 pre-column (100 mm i.d. 5.0 cm length) and a mLC analytical column (75 mm 10 cm) that also served as a mESI emitter were used for the separation of the digested proteins.*
Data format	*Raw*
Experimental factors	*Mouse heart lysates prepared using standard RIPA buffer*
Experimental features	*Immunoprecipitation of fresh mouse heart lysates, captured via Protein-A beads before injection onto mass spectrometry.*
Data source location	*Toronto, Canada*
Data accessibility	Data is within this article and at http://www.proteomexchange.org/*,* reference ID PRIDE: PXD001053, http://proteomecentral.proteomexchange.org/cgi/GetDataset?ID=PXD001053

**Value of the data**

• Control, benchmark data for mouse heart immunoprecipitation.
• Benchmark mass spectrometry data for protein analysis under cardiac specific promoters.
• Comparison data set for heart failure and proteins involved in cardiac function.
• Correlation of mouse heart protein analysis and human iPSC-cardiomyocyte interactions.
• Use of high resolution mass spectrometry on a biological sample with disease phenotype.
• Novel cardiac interactions of ILK, a key protein in cardiac force transduction.
• Only in vivo data available for ILK-expression model, with documented in vivo cardiac phenotype.

## 1 Data

The dataset represents proteins that were identified to interact with integrin-linked kinase after immunoprecipitation from a model of cardiac-specific expression, with identification via mass spectrometry. The RAW data files generated from LC-MS-MS analysis of ILK immunoprecipitations have been provided, and are deposited in the ProteomeExchange (reference ID PRIDE: PXD001053).

## 2 Experimental design, materials and methods

Hearts from ILK-transgenic mice (with cardiac-specific over-expression of ILK wild-type (WT)) were harvested and lysates prepared in ice-cold RIPA buffer [1]. Tissue extracts were prepared with RIPA lysis buffer (150 mM NaCl, 1% Nonidet P40, 05% deoxycholate, 0.1% SDS, 50 mM Tris, pH 8.0 and 1 mM phenylmethylsulphonyl fluoride) supplemented with 1×complete protease inhibitor cocktail (EDTA-free) (Roche Diagnostics GmbH, Penzberg, Germany). The samples were centrifuged, and the resulting protein lysate (2 mg) were incubated overnight at 4 °C with either 2 μg of anti-ILK rabbit antibody (catalogue number: 3856, Cell Signaling Technology) or control rabbit IgG (catalogue number: 011-000-003, Jackson ImmunoResearch Lab. Inc.). The samples were than incubated for 2 h at 4 °C with protein A agarose beads (catalogue number: sc-2001, Santa Cruz Biotechnology) and the bound beads were washed four times with RIPA lysis buffer. The protein samples were supplemented with 1×protein sample buffer, boiled and resolved on a 6-18% acrylamide gradient SDS gel. After Coomassie Blue staining, the extra bands on the IP lane contrasting to the control lane were excised from the gel and tryptically digested for MS/MS analysis. Briefly, excised gel spots were destained and washed with ACN (50%) and AMBIC (100 mM), followed by drying in a vacuum centrifuge (UNIVAPO, uniEquip, Matinsried, Germany). The dried gel pieces were digested with trypsin containing digestion buffer (0.1 μg/μL trypsin, 1 M CaCl_2_, 1 M AMBIC (pH 7.4)) for 45 min on ice. The excess amount of trypsin solution was replaced by the same volume of 100 mM of AMBIC without trypsin and incubated overnight at 37 °C. The peptides were extracted with increasing concentrations of ACN and TFA and dried by vacuum centrifugation before being analyzed by mass spectrometry.

Mass spectrometry was performed using an on-line liquid chromatography-tandem mass spectrometry system. An Agilent 1100 Capillary LC system (Palo Alto, CA, USA) fitted to a LTQ ion trap mass spectrometer (Thermo Electron, San Jose, CA, USA) was used for analysis. A C18 pre-column (100 mm i.d×5.0 cm length) and a μLC analytical column (75 mm×10 cm), that also served as a μESI emitter, were used for the separation of the digested proteins. The mass spectrometer was operated in data-dependent mode, automatically cycling through acquisition of a full-scan mass spectrum and three MS/MS spectra recorded sequentially. A dynamic exclusion list time of 1.5 min was used. For the reverse phase chromatography, an 80-min gradient elution from water to acetonitrile, each containing 0.1% FA and 0.02% TFA, was performed at a flow of 200 nl/min. All MS/MS spectra were searched against the NCBInr protein database using MASCOT Server (v2.2; Matrix Science) [2]. The search results were analysed using the Scaffold software (Proteome Software) [3]. The RAW data files are archived with the ProteomeExchange (address above). Scaffold identified 74 proteins, without a cutoff for the minimum number of spectral counts (range 18 to 1 spectral counts), although two of the proteins identified were likely the antibody used for the immunoprecipitation. To reduce the chance of identifying a non-specific interaction (i.e. “sticky” protein), the results were culled using the CRAPome database [4]. SERCA-2a possessed the largest number of spectral counts, and was fully investigated for biological relevance [5]. Other identified proteins had been investigated as ILK binding partners, including Hsp70, and published [6].
